# Proposal of a grading system for squamous cell carcinoma of the lung — the prognostic importance of tumour budding, single cell invasion, and nuclear diameter

**DOI:** 10.1007/s00428-023-03612-8

**Published:** 2023-08-09

**Authors:** Noémi Zombori-Tóth, Fanni Hegedűs, Szintia Almási, Anita Sejben, László Tiszlavicz, József Furák, Gábor Cserni, Tamás Zombori

**Affiliations:** 1 Csongrád-Csanád County Hospital of Chest Diseases, Deszk, Hungary; 2 Department of Pathology, Albert Szent-Györgyi Medical Centre, University of Szeged, Szeged, Hungary; 3 Department of Surgery, Albert Szent-Györgyi Medical Centre, University of Szeged, Szeged, Hungary; 4 Department of Pathology, Bács-Kiskun County Teaching Hospital, Kecskemét, Hungary

**Keywords:** Lung squamous cell carcinoma, Grade, Tumour budding, Single-cell invasion, Nuclear diameter, Spread through air spaces

## Abstract

The prognostic markers of lung squamous cell carcinoma (LSCC) are less investigated. The aim of our study was to evaluate tumour budding (TB), minimal cell nest size, nuclear diameter (ND), and spread through air spaces (STAS) among patients with resected LSCC, semi-quantitatively. Furthermore, we aimed to identify a grading system for the best prognostic stratification of LSCC. Patients who underwent surgical resection at the Department of Surgery, University of Szeged between 2010 and 2016 were included. Follow-up data were collected from medical charts. Morphological characteristics were recorded from histologic revision of slides. Kaplan-Meier analysis, log rank test and Cox proportional-hazards model, ROC curve analysis, and intraclass correlation were utilised. Altogether 220 patients were included. In univariate analysis, higher degree of TB, infiltrative tumour border, larger ND, the presence of single cell invasion (SCI) and STAS were associated with adverse prognosis. Based on our results, we proposed an easily applicable grading scheme focusing on TB, ND, and SCI. In multivariate analysis, the proposed grading system (*p*_OS _< 0.001, *p*_RFS _< 0.001) and STAS (*p*_OS _= 0.008, *p*_RFS _< 0.001) were independent prognosticators. Compared to the previously introduced grading systems, ROC curve analysis revealed that the proposed grade had the highest AUC values (AUC_OS_: 0.83, AUC_RFS_: 0.78). Each category of the proposed grading system has good (ICC: 0.79–0.88) reproducibility. We validated the prognostic impact of TB, SCI, ND, and STAS in LSCC. We recommend a reproducible grading system combining TB, SCI, and ND for proper prognostic stratification of LSCC patients. Further research is required for validation of this grading scheme.

**Supplementary Information:**

The online version contains supplementary material available at 10.1007/s00428-023-03612-8.

## Introduction

Lung cancer remains the leading cause of cancer mortality worldwide [1], and the second most common cancer following prostate cancer in males, and breast cancer in females [2]. Despite complete surgical resection, the prognosis of lung cancer is generally poor [3], with recurrence rates of 15–30%, and 5-year overall survival (OS) rates of 60–70% [4]. The International Association for the Study of Lung Cancer (IASLC) proposed a prognostic stratification system for lung adenocarcinoma that focuses on tumour growth patterns. This classification was included in the 4th and 5th editions of World Health Organisation (WHO) classification of pulmonary neoplasms [5, 6]. In addition, proliferative index, any amount of solid or micropapillary component, and prominent spread through air spaces (STAS) have shown prognostic potential for lung adenocarcinomas [7–9]. Although several publications have highlighted different prognosticators for pulmonary adenocarcinoma, prognosticators of lung squamous cell carcinoma (LSCC) have not been explored to a similar extent.

LSCC has been categorised into keratinising, non-keratinising, basaloid, and lymphoepithelial types; however, their prognostic implications remain unknown [6, 10]. In LSCC, tumour budding, minimal cell nest size, and nuclear diameter are considered as possible candidates for prognostic purposes [11–13]. Tumour budding is defined as the presence of isolated small tumour nests composed of less than 5 tumour cells at the invasive tumour front [14]. First and foremost, tumour budding was introduced in colorectal cancer as a morphological feature, and its prognostic role has been validated in several publications [15–19]. Internationally accepted reporting and clinical implications were recommended at the International Tumour Budding Consensus Conference in 2016 [20]. Moreover, tumour budding has emerged as a promising prognostic feature in pancreatic adenocarcinoma [21, 22], oral squamous cell carcinoma [23], and cervical squamous cell carcinoma [24], indicating poor survival outcomes and early disease relapse [23, 25, 26].

**Fig. 1:**
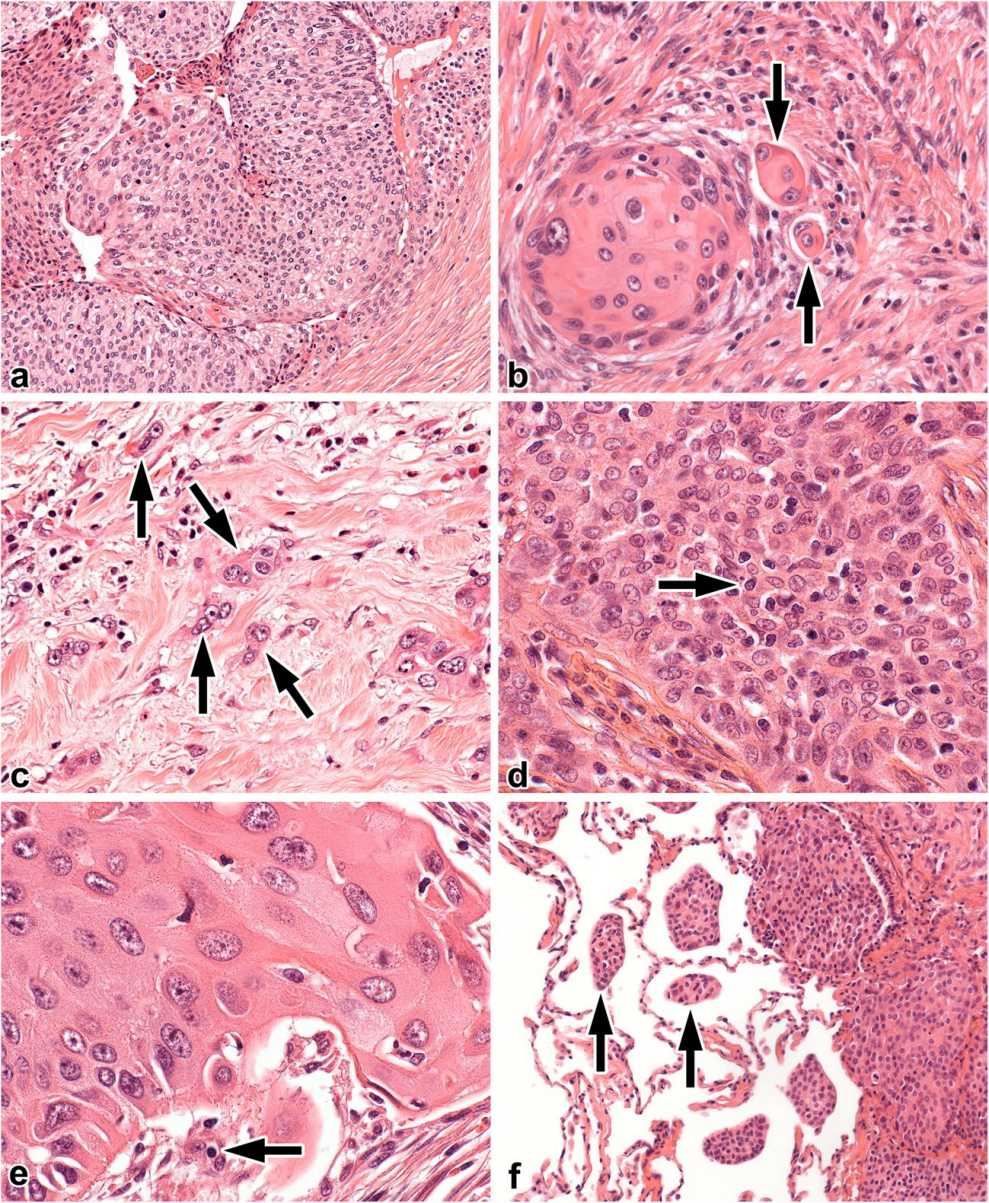
Examples of relevant histologic parameters and their levels. **a** Lack of tumour budding (HE, 100×). **b** Intermediate level of tumour budding (buds – arrows, HE, 400×). **c** High level of tumour budding (buds – arrows, HE, 400×). **d** Squamous cell carcinoma with small nuclear diameter (≤ 4 lymphocytes – arrow, HE, 400×). **e** Squamous cell carcinoma with large nuclear diameter (> 4 lymphocytes – arrow, HE, 400×). **f** Spread through air paces (STAS) in a case of squamous cell carcinoma (STAS – arrows, HE, 100×)

Wankhede et al. demonstrated that the evaluation and statistical interpretation of tumour budding in retrospective studies are controversial in their meta-analysis [27]. Although most authors used haematoxylin eosin (HE) stained slides for the investigation of tumour budding, some others utilised cytokeratin immunostaining, in order to increase the sensitivity of tumour budding identification [28–30]. There are controversies related to the area investigated, and the exact parameter recorded. Most of the publications used 200× magnification (medium power field - MPF), while others utilised 400× (high power field - HPF) [12, 13, 31]. In several studies, only the presence or absence of tumour budding was recorded [30–32]. In other studies, the number of tumour buds was evaluated in one or more power fields; furthermore, the maximum and/or mean number of buds were recorded. When tumour budding was counted, several cut-off values were assessed to identify LSCC subgroups with different prognosis. Figure 1a–c demonstrates different extents of tumour budding. Based on literature data, Wankhede and co-workers identified that the presence of tumour budding has an adverse effect on OS and disease-free survival [27].

The prognostic role of other morphological parameters is less investigated. Minimal cell nest size is defined as the smallest tumour cluster within the tumour or at the invasive front. As Online Resource 1 displays, minimal cell nest size can be subclassified according to the cell number [11–13, 28, 31]. Although Weichert et al. demonstrated that a grading scheme of LSCC focusing on tumour budding and minimal cell nest size is a gender-, age-, and stage independent prognosticator [12], Kadota et al. reported only single cell invasion as an adverse predictor for OS [11].

There are few publications evaluating the nuclear features of LSCC; however, it is an important prognostic factor in other malignancies such as breast cancer or lung adenocarcinoma [33, 34]. The nuclear features of LSCC were extensively investigated by Kadota et al. and nuclear diameter turned out to have a prognostic effect on OS [11, 29]. As Fig. 1d and e represent, the nuclear size is mostly compared to the diameter of the nucleus of a resting lymphocyte [11, 35, 36]. In a subsequent publication, Kadota et al. introduced a new grading system for LSCC combining tumour budding and nuclear diameter [35].

Figure 1f demonstrates spread through air spaces (STAS) in the case of a LSCC. STAS introduced by Kadota et al. is a newly recognised form of invasion in lung cancer [37]. As our previous publications reported, it is the presence is associated with unfavourable outcome in lung adenocarcinomas [8, 9] but its impact on prognosis is less investigated in LSCC. Only Stögbauer et al. and Lu et al. demonstrated that the presence of STAS is an adverse prognostic feature in LSCC [13, 38].

Although evidence is accumulating about prognostic factors of LSCC, there is no internationally accepted grading system for this pulmonary malignancy. The aims of our study were to evaluate semi-quantitatively tumour budding, minimal cell nest size, nuclear diameter, and STAS among patients with resected LSCC. Furthermore, we aimed to identify a grading system for the best prognostic stratification for LSCC.

## Materials and methods

Patients diagnosed with LSCC who underwent surgical resection at the Department of Surgery, University of Szeged, between 2010 and 2016 were included. Exclusion criteria were perioperative death, advanced tumours (pT4, distant metastasis), unavailability of histological slides or clinical follow-up data, and neoadjuvant therapy. The patients’ clinical parameters including age, gender, smoking habits, type of surgery, adjuvant therapy, and follow-up data, namely OS and recurrence-free survival (RFS) were collected from medical charts. All patients had regular follow-up as published previously [9], briefly this consisted of physical examination, chest X-ray, abdominal ultrasonography, and chest computed tomography. The follow-up period ended on July 1, 2022.

Using a multi-headed microscope (Olympus BX43, Tokyo, Japan), 4-μm-thick HE-stained sections from formalin-fixed, paraffin-embedded material were reviewed by three authors (NZT, FH, TZ), who were blinded to clinical outcome of the patients. The following morphological parameters were recorded: histological diagnosis defined by WHO [6], tumour size (mm), distance to resection margin (mm), tumour budding, minimal cell nest size, number of mitosis in 10 HPFs, nuclear diameter, expansive or infiltrative nature of the invasive front, the presence of STAS, vascular, lymphovascular, and pleural invasion. The pT, pN categories, and stages were identified according to the 8th edition of American Joint Committee on Cancer Cancer Staging Manual [39]. In all cases, immunohistochemical reactions, namely thyroid transcription factor-1 (TTF-1), p40 (or p63), and mucin stains were applied to determine the proper histological diagnosis.

Tumour budding was defined as a tumour cell nest with less than 5 cells, surrounded by desmoplastic stroma. Both the presence and the extent of tumour budding were recorded. Regarding the extent, tumour budding was counted with two different methods, published by Kadota et al. [35]. Briefly, the total number of buds on 10 MPFs and the maximum number of buds in one (hot spot) MPF were registered. The degree of tumour budding was classified according to different cut-off points introduced by Kadota et al. and Weichert et al. The former separates low (0–9 buds/10 MPF) and high tumour budding (≥10 buds/10 MPF); meanwhile, Weichert et al. introduced low (0/10 MPF), intermediate (1–14 buds/10 MPF), and high tumour budding (≥15 buds/10 MPF) categories [12, 35]. Tumours with low intermediate and high tumour budding are demonstrated in Fig. 1a–c.

Minimal cell nest size was subdivided into four categories (Online Resource 1), namely large nest (≥15 tumour cells), intermediate nest (5–14 tumour cells), small nest (2–4 tumour cells), and single cell invasion [11–13, 28, 31]. Minimal cell nest size was recorded at the edge of the tumour and in the entire tumour area. The nuclear features, such as nuclear diameter and mitotic activity, were evaluated under HPF (objective: 40×, visible field area = 0.237 mm^2^), based on a method published by Kadota [11, 40, 41]. As Fig. 1d and e display, small nuclear diameter (≤ 4 resting lymphocytes) and large nuclear diameter (> 4 resting lymphocytes) categories were defined [11]. Mitoses were evaluated in 50 HPFs containing the highest mitotic activity and it was calculated for 10 HPFs. Based on previous publications, low mitotic rate (< 15 mitosis/10 HPFs) and high mitotic rate (≥ 15 mitosis/10 HPFs) categories were utilised [11]. STAS was identified if rounded tumour cell nests were present either in the intra-alveolar space or in the bronchiolar system (Fig. 1f). Desquamated ribbons of neoplastic cells or tumour cell nests with jagged edges were defined as artefacts and were excluded from investigation.

Regarding the grading systems of LSCC, Kadota et al. identified low grade (low tumour budding + small or large nuclear diameter), intermediate grade (high tumour budding + small nuclear diameter), and high grade (high tumour budding + large nuclear diameter) categories [35]. Weichert et al. introduced a scoring system focusing on degree of tumour budding (1–3 scores) and minimal cell nest size (1–4 scores), and defined low grade (2–3 scores), intermediate grade (4–6 scores) and high grade (7 scores) categories [12].

Based on preliminary results of receiver operating characteristics (ROC) curve analysis, we introduced a grading system focusing on degree of tumour budding (0–2 scores), the presence of single cell invasion (0–1 score), and large nuclei (0–1 score). Low (cumulative score 0), intermediate (cumulative score: 1–2), and high grades (cumulative score: 3–4) were identified. Table 1 displays the parameters of the proposed grading system.

**Table 1:** Grading proposal for lung squamous cell carcinoma (MPF: mediate power field - 200×)

Extent of tumour budding		Presence of single-cell invasion		Nuclear diameter		Cumulative scores	Grade
0 score	0 bud / 10 MPFs	0 score	Absent	0 score	Small	0 score	Low
1 score	1–14 bud(s) / 10 MPFs	1 score	Present	1 score	Large (> 4 resting lymphocytes)	1–2 score(s)	Intermediate
2 scores	≥15 buds / 10 MPFs					3–4 scores	High

The chi square and Kruskal-Wallis tests were used to identify associations between variables. Univariate Cox proportional hazards model was applied to detect morphological variables having impact on OS and RFS. Those found significant in the univariate analysis were entered into multivariate Cox proportional hazards model. To avoid statistical bias in multivariate regressions, the overlapping parameters (e.g. tumour budding, minimal cell nest size) were excluded from each model. ROC curve analysis was applied to determine the best variation of parameters included in the proposed grading system. Intraclass correlation coefficient (ICC: two-way mixed effects, absolute agreement, single rater) was applied to measure inter-observer variability of tumour budding, single cell invasion, nuclear diameter, and the categories of proposed grading system. The ICC inter-rater agreement measures defined by Koo and Li [42] were utilised. Statistical models were fitted using SPSS Statistics V 23.0 software (Armonk, USA). Our study was approved by the institutional ethical committee of the Albert Szent-Györgyi Clinical Centre of the University of Szeged.

## Results

Altogether 912 patients diagnosed with lung cancer were operated on at the Department of Surgery, University of Szeged between 2010 and 2016. LSCC was detected in 252 cases, adenocarcinoma was diagnosed in 524, and others (sarcomatoid carcinoma, neuroendocrine neoplasms etc.) were found in 136 cases. Due to neoadjuvant therapy, multiple tumours, or absence of follow-up data, 32 patients with LSCC were excluded. Overall 220 patients were included in our study. Median age was 63.8 years (range: 43.7–83.5 years). The relationships among clinical characteristics and tumour budding, single cell invasion, and nuclear diameter are displayed in Online Resource 2. The presence of tumour budding was associated with smoking history (*p *= 0.003) and with higher stage (*p *= 0.031), while single cell invasion was significantly more frequent in cases with higher nodal status (*p *< 0.001) and with higher stage (*p *< 0.001). The relation of morphological parameters with tumour budding, single cell invasion and nuclear diameter are demonstrated in Table 2. Tumour budding was recorded mostly in keratinising histological subtype (58%). Tumour budding was associated with infiltrative tumour border (*p *< 0.001), smaller minimal cell nest size categories (*p *< 0.001), single cell invasion (*p *< 0.001), larger nuclear diameter (*p *= 0.023), pleural (*p *= 0.021), vascular (*p *= 0.006), and lymphovascular invasion (*p *< 0.001). Single cell invasion was related to infiltrative tumour border (*p *< 0.001), smaller minimal cell nest size categories (*p *< 0.001), vascular (*p *= 0.05), and lymphovascular invasion (*p *< 0.001). Finally, large nuclear diameter was found to be more frequent in smaller minimal cell nest size categories (*p *= 0.035).

**Table 2:** Associations between morphological characteristics and tumour budding, single-cell invasion and nuclear diameter (STAS: spread through air spaces, NA: not applicable)

Parameters	Absence of tumour budding	Presence of tumour budding	*p*	Absence of single-cell invasion	Presence of single-cell invasion	*p*	Small nuclear diameter	Large nuclear diameter	*p*
	*n*			*n*			*n*		
Histological subtype			<0.001*			0.325*			0.009*
Keratinising	29	83		56	56		67	45	
Non-keratinising	39	52		51	40		70	21	
Basaloid	10	7		10	7		13	4	
Tumour budding			NA			**<0.001**			0.023
Absent	78			68	10		61	17	
Present		142		49	93		89	53	
Type of invasive border			**<0.001**			**<0.001**			0.073
Pushing margin	69	91		101	59		115	45	
Infiltrative margin	9	51		16	44		35	25	
Minimal cell nest size			<0.001*			<0.001*			0.035*
Single cell	10	93		0	103		64	39	
Small	0	49		45	0		30	15	
Intermediate	20	0		22	0		17	5	
Large	48	0		50	0		39	11	
Single-cell invasion			**<0.001**			NA			0.082
Absent	68	49		117			86	31	
Present	10	93			103		64	39	
Nuclear diameter			**0.023**			0.082			NA
Small	61	89		86	64		150		
Large	17	53		31	39			70	
Mitotic activity						0.879			0.063
Low	23	29	0.139	27	25		41	11	
High	55	113		90	78		109	59	
Pleural invasion			0.021*			0.097*			0.412*
PL0	74	120		107	87		134	60	
PL1	2	0		0	5		0	3	
PL2	1	9		6	1		8	1	
PL3	1	13		4	10		8	6	
Vascular invasion			**0.006**			**0.05**			0.267
Absent	72	107		102	77		126	53	
Present	7	34		16	25		25	16	
Lymphovascular invasion			**<0.001**			**<0.001**			0.560
Absent	53	51		72	32		74	30	
Present	26	90		46	70		77	39	
Perineural invasion			0.122			0.097			0.434
Absent	71	114		104	81		129	56	
Present	8	27		14	21		22	13	
STAS			0.865			0.891			0.484
Absent	63	100		92	81		120	53	
Present	16	41		25	22		30	17	

*Kruskal-Wallis tests, others chi-square tests

Bold values denote statistical significance at the *p* < 0.05 level

Altogether recurrence was detected in 54 patients. Most of them had intrathoracic recurrence (*n *= 45, 83%). Extrathoracic recurrence (*n *= 9, 17%) included liver, adrenal, bone, and brain metastases. Thirty patients (13.6%) died from either progression of LSCC or other causes. The median RFS and OS estimates were 19.3 months (range: 1.9–127.5 months) and 23.0 months (range: 2.1–73.8 months), respectively. The median follow-up was 81 months (range: 1.9–138 months).

Online Resource 3 displays the results of univariate analysis of clinical parameters. Neither of them played significant role in the prognosis. Table 3 demonstrates the results of univariate analysis of morphological factors. In univariate analysis of OS, the presence and higher degree of tumour budding, infiltrative tumour border, single-cell invasion, large nuclear diameter, higher Kadota-grade, higher Weichert-grade, the presence of STAS, higher pT, pN categories and higher stage were associated with adverse prognosis. In univariate analysis of RFS estimates, infiltrative tumour border, smaller categories of minimal cell nest size, the presence of single-cell invasion, large nuclear diameter, higher Kadota-grade, higher Weichert-grade, the presence of STAS, higher pT, pN categories, and higher stage had an adverse impact on prognosis.

**Table 3:** Results of univariate Cox proportional hazards model (OS: overall survival, RFS: recurrence-free survival, HR: hazard ratio, CI: confidence interval, STAS: spread through air spaces)

Parameters	Overall survival			Recurrence-free survival		
	HR	95% CI	*p*	HR	95% CI	*p*
Histological subtype						
Keratinising	Reference			Reference		
Non-keratinising	**0.43**	**0.19–0.98**	**0.046**	0.57	0.32**–**1.03	0.063
Basaloid	0.28	0.03**–**2.10	0.283	0.56	0.17**–**1.82	0.336
Tumour budding						
Absent	Reference			Reference		
Present	**5.71**	**1.72–18.89**	**0.004**	1.61	0.89**–**2.90	0.111
Tumour budding extension (Weichert [12])						
Low	Reference			Reference		
Intermediate	**4.98**	**1.46–16.95**	**0.010**	1.48	0.79**–**2.76	0.217
High	**8.09**	**2.18–29.95**	**0.002**	2.06	0.95**–**4.47	0.065
Type of tumour edge						
Pushing margin	Reference			Reference		
Infiltrative margin	**3.99**	**1.94–8.23**	**<0.001**	**2.35**	**1.37–4.03**	**0.002**
Minimal cell nest size						
Single cell	Reference			Reference		
Small	0.73	0.31**–**1.72	0.481	**0.438**	**0.20–0.94**	**0.036**
Intermediate	0.37	0.22**–**1.61	0.190	0.47	0.18**–**1.21	0.119
Large	0.12	0.12**–**1.34	0.671	**0.29**	**0.12–0.66**	**0.003**
Single-cell invasion						
Absent	Reference			Reference		
Present	**2.99**	**1.36–6.55**	**0.006**	**2.65**	**1.51–4.64**	**0.001**
Nuclear diameter						
Small	Reference			Reference		
Large	**5.94**	**2.72–13.0**	**<0.001**	**3.67**	**2.14–6.29**	**<0.001**
Kadota-grade [35]						
Low	Reference			Reference		
Intermediate	0.24	0.03**–**1.78	0.162	0.45	0.16**–**1.28	0.137
High	**6.73**	**3.09–14.63**	**<0.001**	**3.79**	**1.88–7.64**	**<0.001**
Weichert-grade [12]						
Low	Reference			Reference		
Intermediate	**6.04**	**1.40–26.00**	**0.016**	**2.40**	**1.17–4.9**	**0.016**
High	**11.86**	**2.55–55.08**	**0.002**	**3.56**	**1.50–8.54**	**0.004**
Grading proposal in present publication						
Low	Reference			Reference		
Intermediate	**7.01**	**1.10–53.57**	**0.045**	**4.58**	**1.61–13.10**	**0.004**
High	**20.00**	**2.64–74.32**	**<0.001**	**8.80**	**2.98–25.91**	**<0.001**
STAS						
Absent	Reference			Reference		
Present	**2.63**	**1.26–2.63**	**0.009**	**3.32**	**1.92–5.74**	**<0.001**
pT						
pT1	Reference			Reference		
pT2	**1.24**	**1.01–5.32**	**0.003**	**1.98**	**1.14–6.58**	**0.045**
pT3	**2.34**	**1.3–12.47**	**0.008**	**3.21**	**1.4–16.56**	**<0.001**
pN						
pN0	Reference			Reference		
pN1	1.73	0.78**–**2.15	0.126	2.1	0.85**–**4.88	0.109
pN2	**3.24**	**1.29–7.67**	**0.023**	**3.87**	**1.50–8.34**	**0.030**
Stage						
I	Reference			Reference		
II	1.56	0.88**–**4.23	0.140	2.04	0.91**–**5.46	0.161
III	**3.01**	**1.29–9.34**	**0.002**	**3.65**	**1.4–14.99**	**<0.001**

Bold values denote statistical significance at the *p* < 0.05 level

**Fig. 2:**
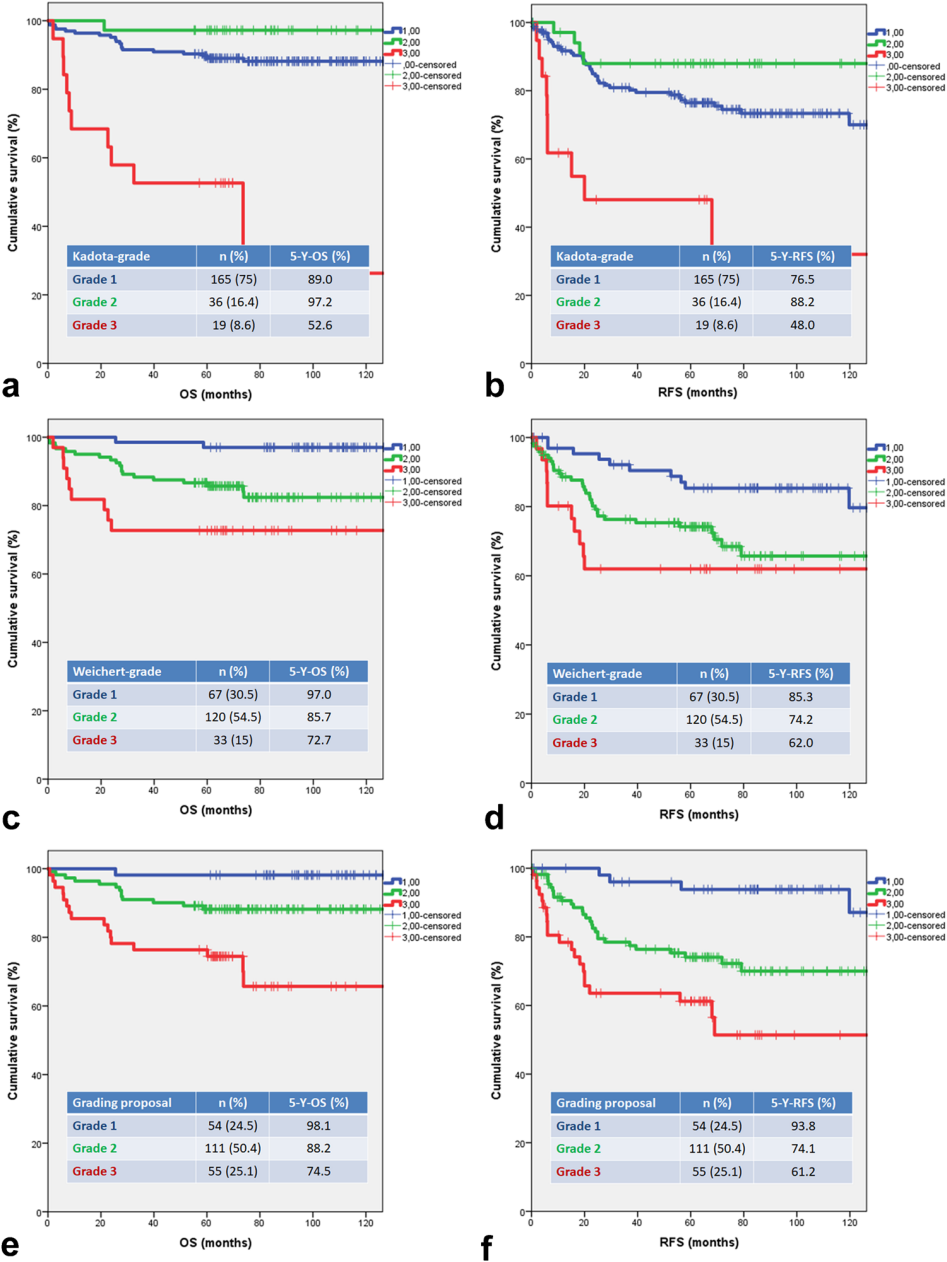
Kaplan-Meier curves for OS and RFS according to different grading systems. **a**, **b** Regarding the grade defined by Kadota et al, significant differences were demonstrated among OS and RFS estimates of G1 vs.G3 (*p*_OS _< 0.001; *p*_RFS _< 0.001) and G2 vs G3 *p*_OS _< 0.001; *p*_RFS_<0.001), but not between G1 vs. G2 (*p*_OS _= 0.131; *p*_RFS_ = 0.128). **c**, **d** Concerning the grade introduced by Weichert et al., significant differences were found among OS and RFS estimates of G1 vs. G2 (*p*_OS _= 0.006; *p*_RFS_ = 0.010) and G1 vs. G3 (*p*_OS _< 0.001, *p*_RFS _= 0.004), but not between G2 vs. G3 (*p*_OS _= 0.066; *p*_RFS _= 0.275). **e**, **f** Regarding the proposed grade combining tumour budding, nuclear diameter and single cell invasion, the Kaplan-Meier estimation revealed significant differences among OS and RFS estimates of all grades (G1 vs. G2: *p*_OS _= 0.035; *p*_RFS _< 0.001; G1 vs. G3 *p*_OS _< 0.001; *p*_RFS _< 0.001; G2 vs. G3 *p*_OS _= 0.003; *p*_RFS _= 0.014)

As Fig. 2a–d demonstrate, the two aforementioned grading schemes failed to separate the three prognostic categories. Therefore, we aimed to compose a grade stratifying the patients properly according to the prognosis. Online Resource 4 demonstrates the results of ROC curve analysis. The combining of tumour budding, single-cell invasion, and nuclear diameter resulted in a prognosticator with the highest area under curve (AUC) values (AUC_OS_: 0.83, AUC_RFS_: 0.76). Based on these results, we proposed an easily applicable prognostic system, which combines tumour budding, single-cell invasion, and nuclear diameter. Figure 2e and f present that significant differences were found among OS and RFS estimates of all categories of the proposed grading system.

Comparing to grading systems published by Kadota et al. and Weichert et al. with our proposed grading system in ROC curve analysis, the latter one had the highest AUC value (regarding OS: AUC_proposed grade_: 0.83, AUC_Kadota_: 0.60, AUC_Weichert_: 0.68, regarding RFS: AUC_proposed grade_: 0.78, AUC_Kadota_: 0.52, AUC_Weichert_: 0.59). Online resource 5 displays the results of the multivariate Cox hazard proportional models of OS and RFS, respectively. Among the findings, we underline that the proposed grading system and STAS were independent prognostic markers in our cohort (see Online resource 5: OS – Regression V and RFS – Regression III). Concerning the reproducibility of the proposed grading scheme, the ICC revealed that each parameter, including the categories of grading scheme proposed, has a good (ICC: 0.79–0.88) reproducibility (see Online resource 6).

## Discussion

Despite the fact that LSCC is a frequent primary lung neoplasm, it is less investigated. We aimed to analyse the prognostic impact of different morphological characteristics in a relatively large population of patients diagnosed with resected LSCC. Concerning our results, we focused on tumour budding, nuclear diameter, minimal cell nest size, and STAS.

Tumour budding represents isolated small tumour nests, composed of less than 5 tumour cells at the invasive tumour front (Fig. 1b, c). Tumour budding is a morphological pattern of tumour invasion associated with unfavourable prognosis in different carcinomas, namely colorectal adenocarcinoma [15–19], pancreatic adenocarcinoma [21, 22], oral squamous cell carcinoma [23], and cervical squamous cell carcinoma [24]. In colorectal adenocarcinoma, the counting of buds in 10 HPFs was proposed to be a more reliable and reproducible method than the bud detection in 1 HPF (hot spot) [14]. Tumour budding has been recently identified as a poor prognostic factor in LSCC and in lung adenocarcinoma [11, 30, 43], as well. Furthermore, not only the presence, but the greater extent of tumour budding was associated with adverse prognosis [12, 35]. Kadota et al. and Weichert et al. identified tumour budding categories in 1 MPF and in 10 MPFs with different OS [12, 35] and RFS estimates [35]. Keeping with the aforementioned results, the greater extent of tumour budding was associated with unfavourable OS and RFS estimates in our study.

Grading systems focusing on nuclear features were established in breast, kidney, and bladder carcinoma [44–46]. Moreover, this has also been investigated in lung adenocarcinomas [41]; however, its prognostic impact is less evaluated in LSCC. Kadota et al. reported that nuclear atypia (pleomorphism) was not statistically significant for predicting prognosis in LSCC [35]; however, large nuclei were significantly associated with a worse OS estimate [35]. Therefore, small nuclear diameter (≤ 4 resting lymphocytes) and large nuclear diameter (> 4 resting lymphocytes) categories were defined (Fig. 1d, e). On the other hand, Weichert et al. did not find association between nuclear diameter and prognosis [12]. Similarly to the results of Kadota et al., patients with large nuclei were independently associated with worse OS and RFS estimates. In contrast to the unfavourable prognostic value of a higher mitotic count in lung adenocarcinomas [41], the prognostic impact of higher mitotic count is still controversial in LSCC [35]. In keeping with the results of Kadota et al., the mitotic count did not show any association with clinical outcome in our cohort.

Minimal cell nest size is defined as the smallest cluster of tumour cells surrounded by tumour stroma. As Online resource 1 demonstrates, minimal cell nest size has four distinct categories, namely large, intermediate, small cell nest size, and single-cell invasion. Weichert et al. found that the higher OS estimates were detected in patients with large cell nest size while decreased OS estimates were associated with single-cell invasion [12]. Kadota et al. also assessed the size of the tumour nest and they reported that the smallest tumour nest, namely single-cell invasion was an independent prognostic factor [11]. Correspondingly with the results of the aforementioned publications, single-cell invasion was proven as an adverse prognosticator for both OS and RFS in our cohort.

The recently described form of invasion, namely STAS, has been reported in primary and secondary lung neoplasms. STAS represents rounded tumour cell nests mostly in the intra-alveolar space (Fig. 1f). Its prognostic role is well investigated in lung adenocarcinomas; furthermore, the presence of STAS in LSCC was associated with unfavourable outcome [38]. According to our experience, STAS in not a frequent phenomenon in LSCC; however, in keeping with the results of others [38], patients having STAS had poorer prognosis. As Online resource 7 demonstrates, a mimic of STAS is the endoalveolar spread of squamous cell carcinoma. In this case, the neoplastic squamous epithelium grows along the alveolar septa, protrudes into the lumen and these intraluminal tufts are covered by pneumocytes. Although, endoalveolar spread seems to be similar to STAS, the pneumocyte covering assumes a more cohesive structure. The prognostic role of endoalvelar spread needs further investigations.

Grade is an important prognostic feature of cancers; it influences therapeutic decisions, and it is a standard parameter in the stratification of patients for clinical trials [35]. For example, in breast cancer, the histological grade based on three morphological features provides a strong predictor of outcome [47]. In LSCC, Kadota et al. and Weichert et al. have recently proposed grading schemes. The former workgroup combined tumour budding and nuclear diameter, while the latter one combined tumour budding and minimal tumour cell nest size [12, 35]. The system defined by Weichert et al. utilises similar architectural parameters, because tumour budding and the categories of tumour cell nest size are defined by the number of tumour cells within the tumour cell clusters. Furthermore, the definition of tumour bud and small cell nest size are the same. As our results demonstrate, both grading systems have significant prognostic roles among patients with LSCC. However, there were no significant differences between Kadota-grade 1 vs. grade 2, and between Weichert-grade 2 vs. grade 3, respectively (Fig. 2a–d).

Based on our results, tumour budding, single-cell invasion, and nuclear diameter have an impact on clinical outcome. Therefore, we propose a grading system which includes these three histomorphological parameters in order to identify properly the prognosis of patients with LSCC. In ROC curve analysis, we compared the proposed grading system with the grading schemes published by Kadota et al. [35] and Weichert et al. [12]. According to our results, the proposed grading scheme was superior to others regarding the clinical outcome.

There are certain limitations in our study. First of all, our investigation is a retrospective study. There was no opportunity to evaluate the rare subtypes of LSCC, namely basaloid squamous cell carcinoma and lymphoepithelial carcinoma. Altogether 17 patients diagnosed with basaloid squamous cell carcinoma were included in our evaluation. However, our results demonstrated neither better, nor poorer prognosis of this tumour, more investigations are required to address the prognostic role of basaloid histology. Further limitation is that the proposed grading scheme has to be validated in the future in different, larger cohorts.

Concerning the strengths of our study of consecutive cases, this is the first investigation aiming at the prognostic validation of the grading systems for LSCC published by Kadota et al. and Weichert et al. However, both grading systems had prognostic roles, the two aforementioned grading schemes failed to separate the three prognostic categories. Therefore, we proposed an alternative, easily applicable, and reproducible grading system combining the most important prognostic parameters. Furthermore, a relatively large cohort of patients was evaluated and the median follow-up was longer than 5 years. In addition, we used relatively rigorously the definitions of the morphological parameters.

In conclusion, we validated the prognostic impact of recently introduced morphological parameters, namely tumour budding, single-cell invasion, nuclear diameter, and STAS in LSCC. For the first time, the grading schemes introduced by Weichert et al. and Kadota et al. were validated, as well. We proposed a combined grading system focusing on tumour budding, single-cell invasion, and nuclear diameter for having a proper prognostic stratification in LSCC. Further research is required for validation of the proposed grading scheme, and gathering more data about prognostic markers of LSCC.

### Supplementary information

Online resource 1 The categories of minimal cell nest size: **a** Large cell nest size - nest with ≥15 tumour cells (HE, 200x). **b** Intermediate cell nest size - nest with 5-14 tumour cells (HE, 200x). **c** Small cell nest size - nest with 2-4 tumour cells (HE, 400x). **d** Single-cell invasion (HE, 630x). (PNG 6555 kb)

High Resolution (TIF 8980 kb)

Online resource 2 (DOCX 20 kb)

Online resource 3 (DOCX 16 kb)

Online resource 4 The results of receiver operating characteristic (ROC) curve analysis of variables regarding overall survival (**a**) and recurrence-free survival (**b**). (PNG 570 kb)

High Resolution (TIF 795 kb)

Online resource 5 (DOCX 16 kb)

Online resource 6 (DOCX 13 kb)

Online resource 7 Endoalveolar spread of squamous cell carcinoma. The neoplastic epithelium grows along alveolar septa, protrudes into the lumen and these neoplastic tufts are covered by non-neoplastic pneumocytes (a HE, 100x, b HE, 400x). (PNG 3576 kb)

High Resolution (TIF 4786 kb)

## Data Availability

The data that support the findings of this study are not openly available due to reasons of sensitivity and are available from the corresponding author upon reasonable request. Data is located in controlled access data storage at University of Szeged.
