# Supplementary figures and images for: Proposal of a grading system for squamous cell carcinoma of the lung — the prognostic importance of tumour budding, single cell invasion, and nuclear diameter
Source: Virchows Arch. 2023 Aug 9;483(3):393–404. doi: 10.1007/s00428-023-03612-8 (PMC10542270; doi:10.1007/s00428-023-03612-8)

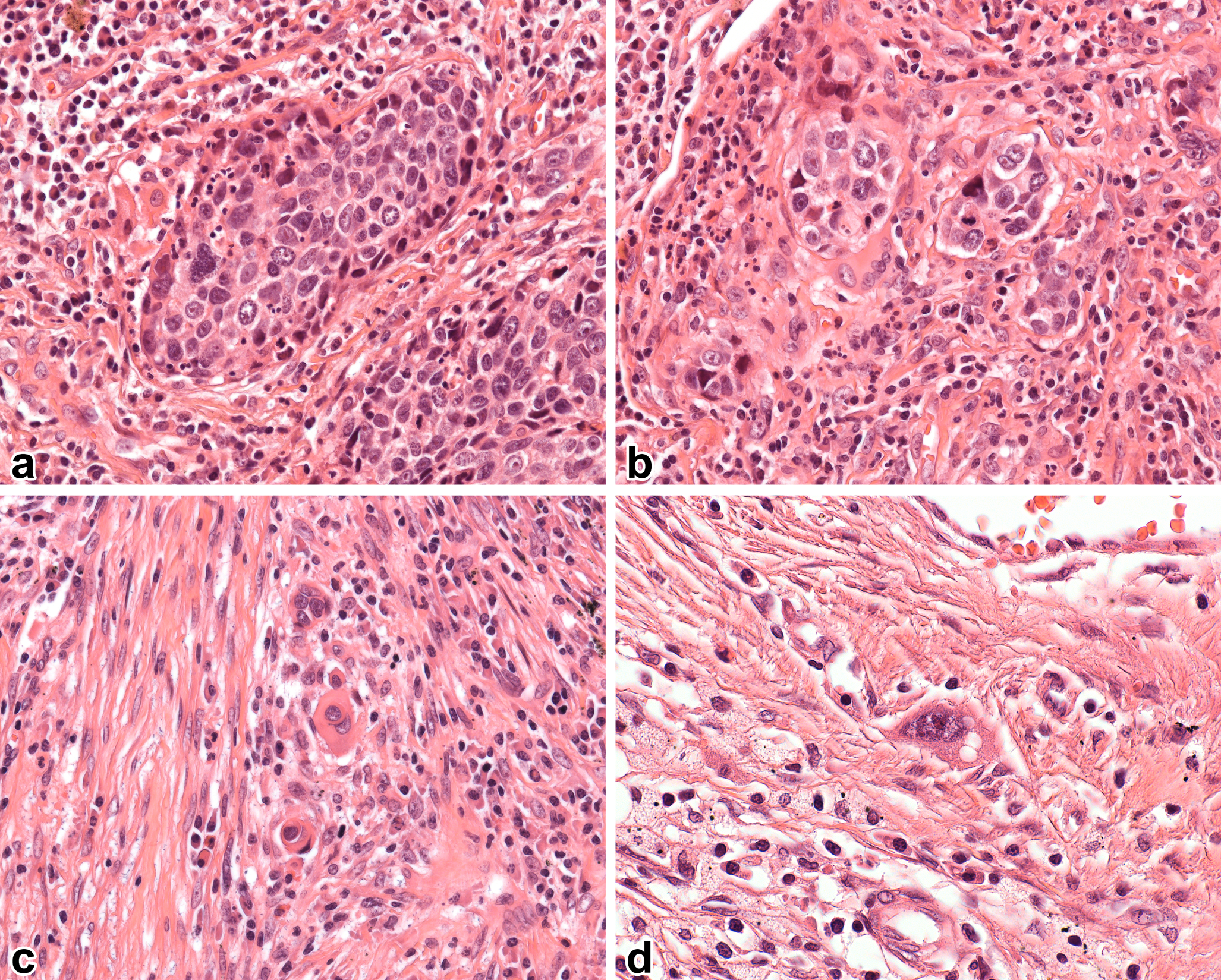

Supplement: Supplementary file 1 — The categories of minimal cell nest size: a Large cell nest size - nest with ≥15 tumour cells (HE, 200x). b Intermediate cell nest size - nest with 5-14 tumour cells (HE, 200x). c Small cell nest size - nest with 2-4 tumour cells (HE, 400x). d Single-cell invasion (HE, 630x). (PNG 6555 kb) [file 428_2023_3612_Fig3_ESM.png]

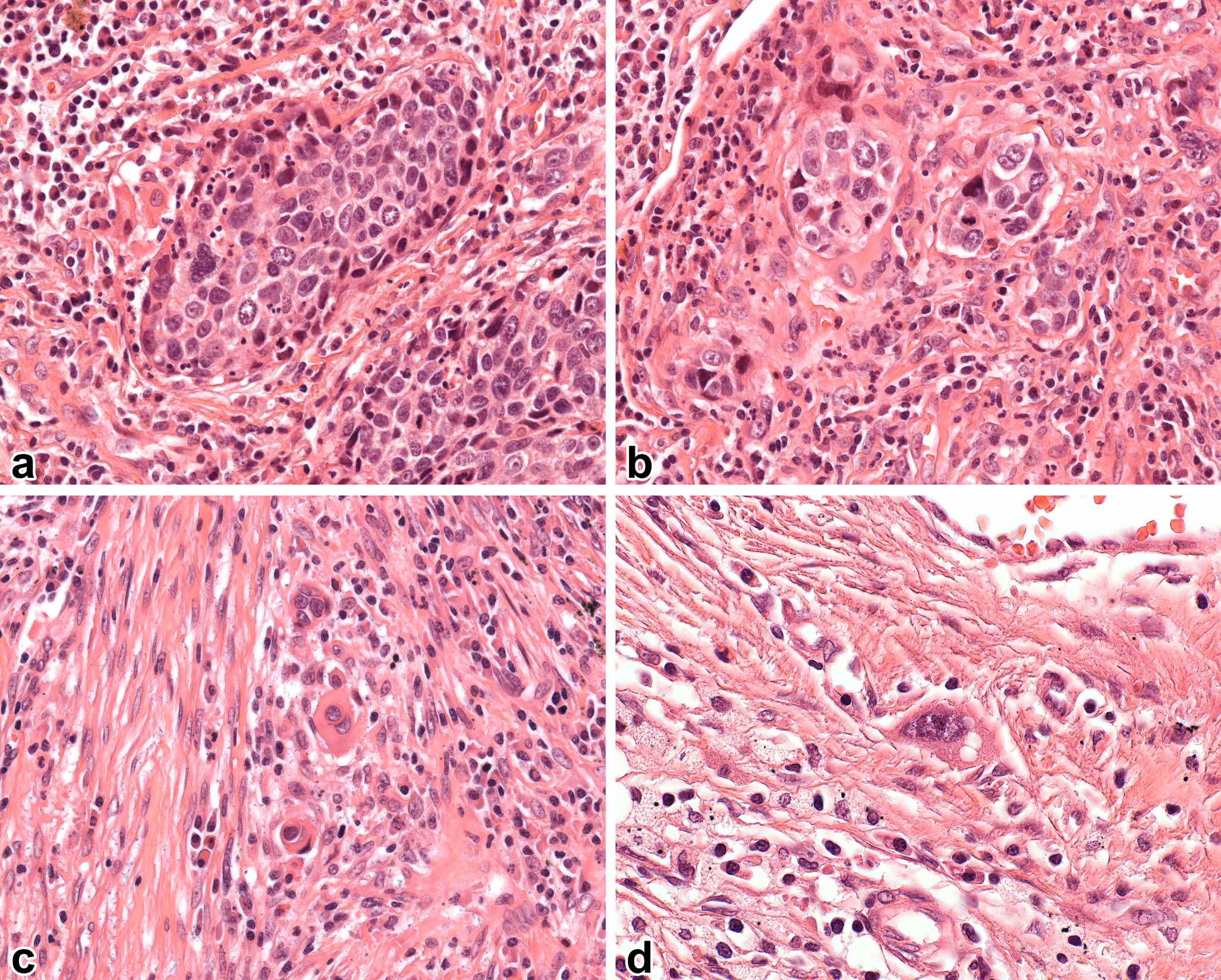

Supplement: Supplementary file 2 — High Resolution (TIF 8980 kb) [file 428_2023_3612_MOESM1_ESM.tif]

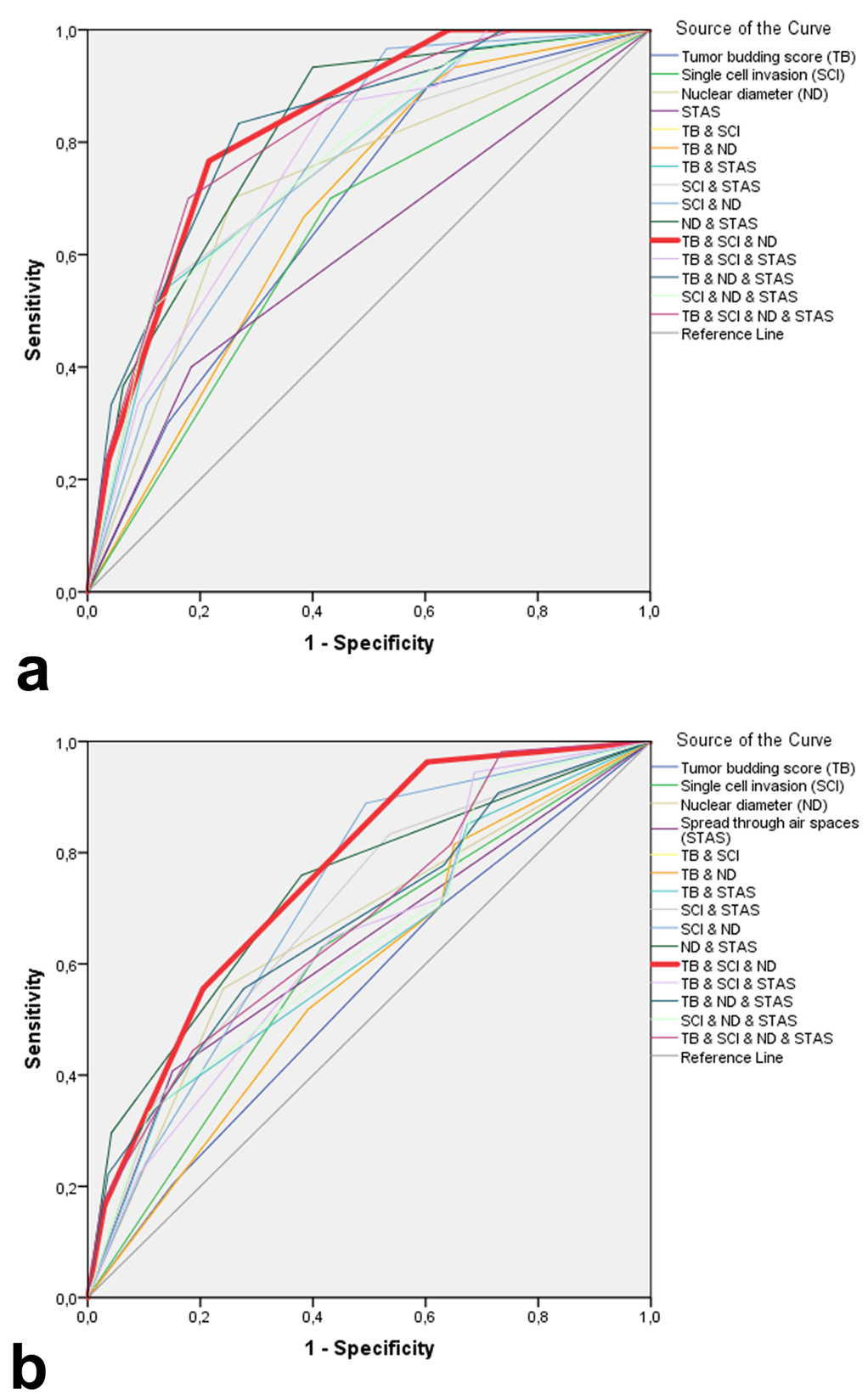

Supplement: Supplementary file 5 — The results of receiver operating characteristic (ROC) curve analysis of variables regarding overall survival (a) and recurrence-free survival (b). (PNG 570 kb) [file 428_2023_3612_Fig4_ESM.png]

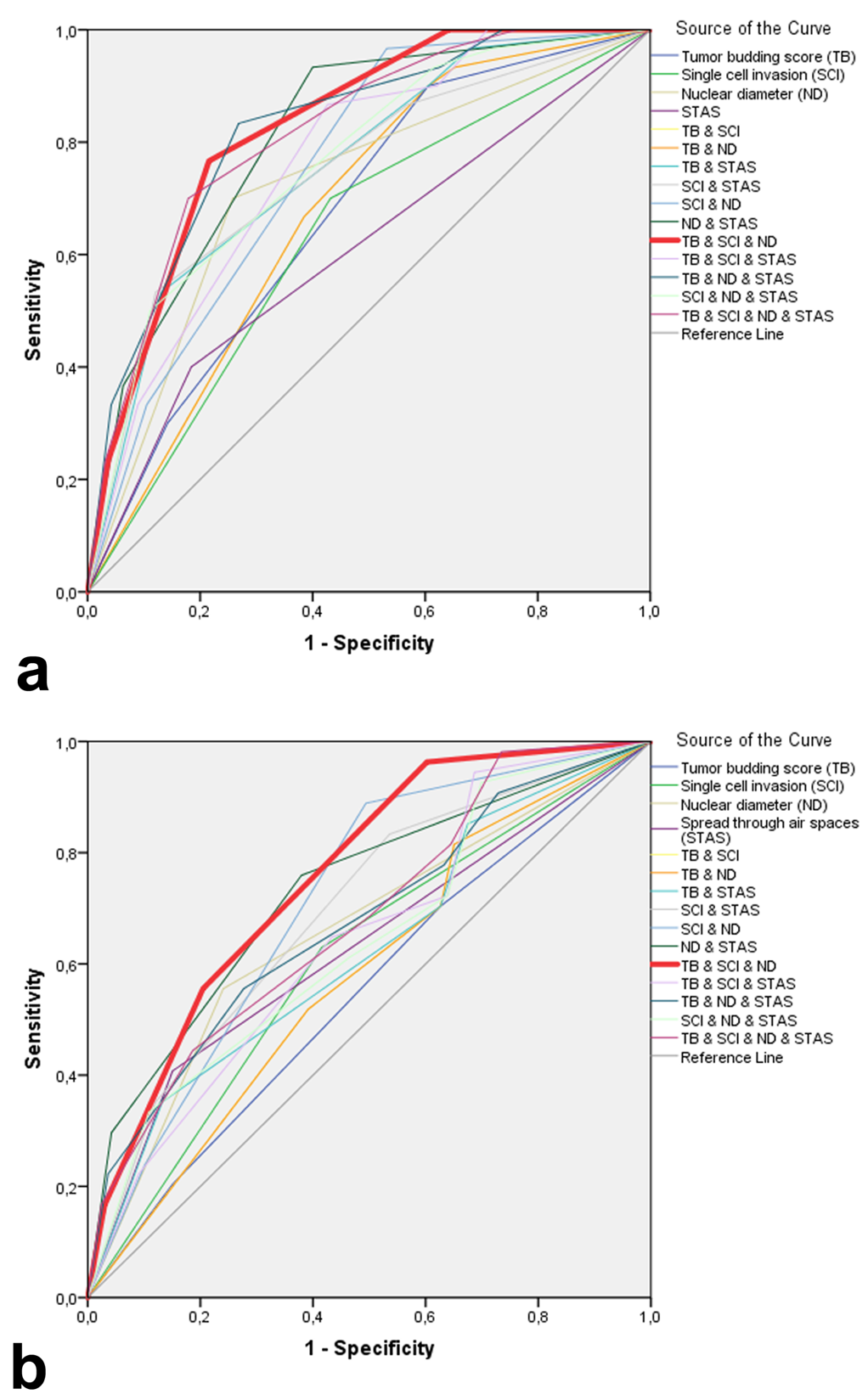

Supplement: Supplementary file 6 — High Resolution (TIF 795 kb) [file 428_2023_3612_MOESM4_ESM.tif]

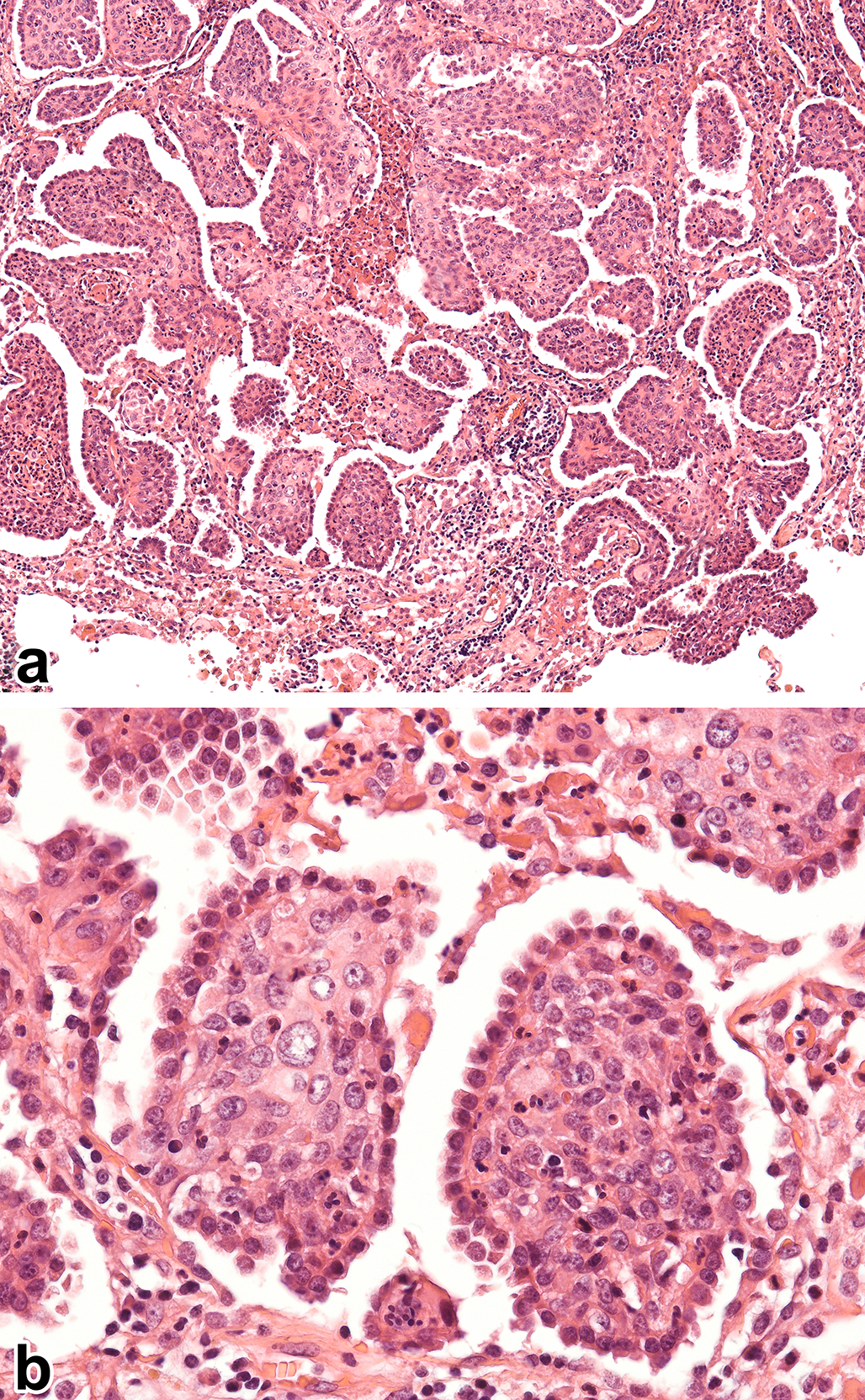

Supplement: Supplementary file 9 — Endoalveolar spread of squamous cell carcinoma. The neoplastic epithelium grows along alveolar septa, protrudes into the lumen and these neoplastic tufts are covered by non-neoplastic pneumocytes (a HE, 100x, b HE, 400x). (PNG 3576 kb) [file 428_2023_3612_Fig5_ESM.png]

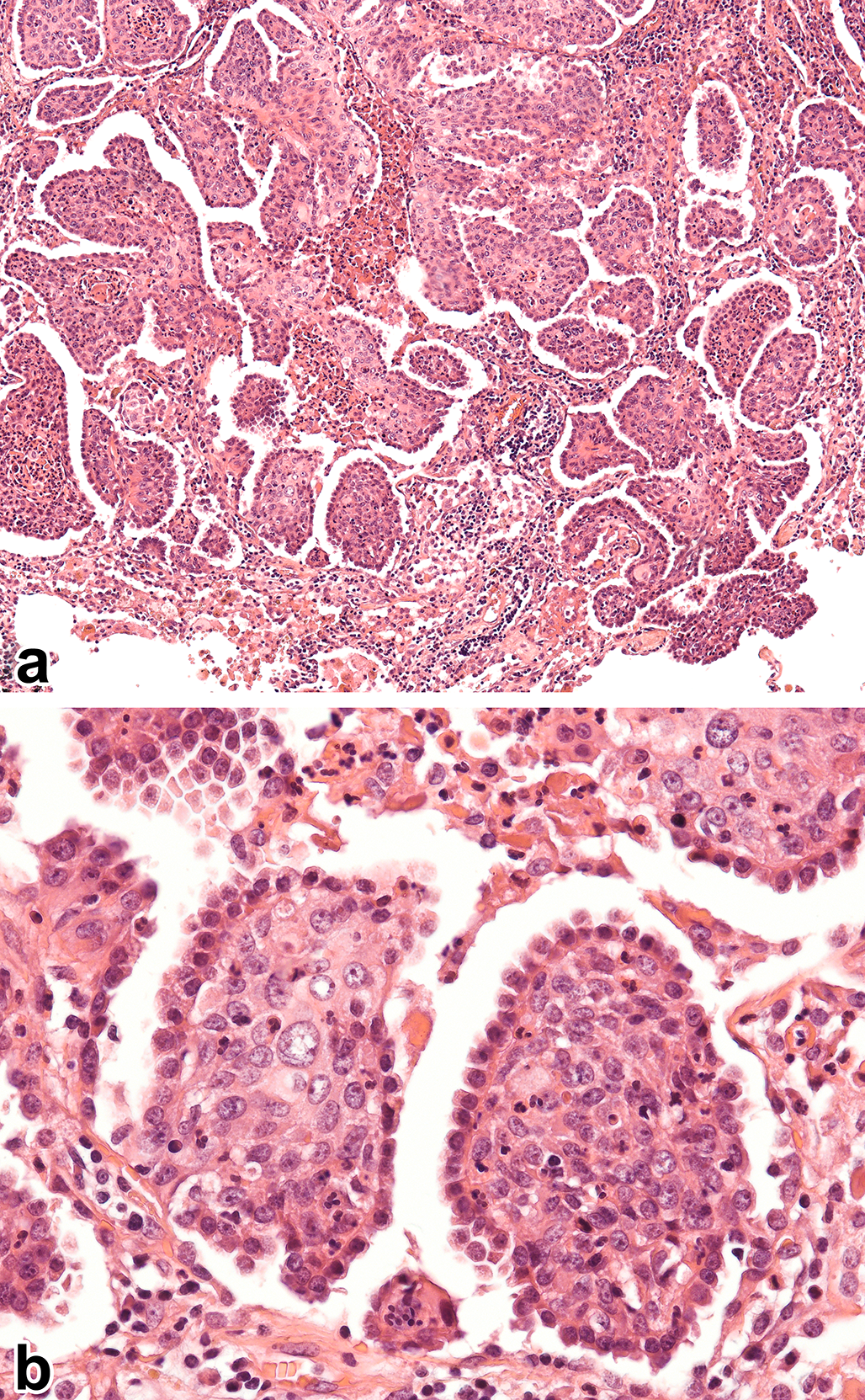

Supplement: Supplementary file 10 — High Resolution (TIF 4786 kb) [file 428_2023_3612_MOESM7_ESM.tif]
