# Supplementary material for: Proposal of a grading system for squamous cell carcinoma of the lung — the prognostic importance of tumour budding, single cell invasion, and nuclear diameter
Source: Virchows Arch. 2023 Aug 9;483(3):393–404. doi: 10.1007/s00428-023-03612-8 (PMC10542270; doi:10.1007/s00428-023-03612-8)
Supplement: Supplementary file 3 — (DOCX 20 kb) [file 428_2023_3612_MOESM2_ESM.docx]

Article title: Proposal of a grading system for squamous cell carcinoma of the lung – the prognostic importance of tumour budding, single cell invasion, and nuclear diameter

Journal name: Virchows Archiv

Author names: Noémi Zombori-Tóth, Fanni Hegedűs, Szintia Almási, Anita Sejben, László Tiszlavicz, József Furák, Gábor Cserni, Tamás Zombori

Corresponding author: Tamás Zombori, MD, PhD; [zombori.tamas@med.u-szeged.hu](mailto:zombori.tamas@med.u-szeged.hu)

**Online Resource 2** Associations between clinical characteristics and tumour budding, single cell invasion and nuclear diameter (STAS: spread through air spaces)

|  | Absence of tumour budding | Presence of tumour budding | *p* | Absence of single cell invasion | Presence of single cell invasion | *p* | Small nuclear diameter | Large nuclear diameter | *p* |
| --- | --- | --- | --- | --- | --- | --- | --- | --- | --- |
| Parameters | n | |  | n | |  | n | |  |
| Age (years) |  |  |  |  |  | 0.68 |  |  | 0.251 |
| <64 | 44 | 69 | 0.32 | 62 | 51 |  | 73 | 40 |  |
| ≥64 | 34 | 73 |  | 55 | 52 |  | 77 | 30 |  |
| Gender |  |  |  |  |  | 0.55 |  |  | **0.026** |
| Female | 28 | 35 | 0.08 | 36 | 27 |  | 50 | 13 |  |
| Male | 50 | 107 |  | 81 | 76 |  | 100 | 57 |  |
| Smoking |  |  | **0.003** |  |  | 0.575 |  |  | 0.687 |
| Never | 18 | 7 |  | 16 | 9 |  | 17 | 8 |  |
| Ever | 60 | 135 |  | 101 | 94 |  | 133 | 62 |  |
| Surgery |  |  |  |  |  | 0.477 |  |  | 0.870 |
| Sublobar resection | 8 | 30 | **0.042** | 18 | 20 |  | 26 | 12 |  |
| Lobectomy | 70 | 112 |  | 99 | 83 |  | 124 | 58 |  |
| Adjuvant therapy |  |  | 0.877 |  |  | 0.304 |  |  | 0.751 |
| Absent | 56 | 99 |  | 86 | 69 |  | 107 | 48 |  |
| Present | 22 | 43 |  | 31 | 34 |  | 43 | 22 |  |
| pT |  |  | 0.311* |  |  | 0.115* |  |  | 0.063 |
| pT1 | 35 | 54 |  | 53 | 35 |  | 67 | 21 |  |
| pT2 | 29 | 52 |  | 39 | 42 |  | 50 | 31 |  |
| pT3 | 14 | 36 |  | 25 | 26 |  | 33 | 18 |  |
| pN |  |  | 0.087* |  |  | **0.001*** |  |  | 0.991 |
| pN0 | 57 | 83 |  | 88 | 52 |  | 94 | 46 |  |
| pN1 | 10 | 31 |  | 16 | 25 |  | 27 | 14 |  |
| pN2 | 12 | 27 |  | 15 | 24 |  | 27 | 12 |  |
| Stage |  |  | **0.031*** |  |  | **0.001*** |  |  | 0.767 |
| I | 40 | 47 |  | 58 | 29 |  | 59 | 28 |  |
| II | 20 | 55 |  | 35 | 40 |  | 50 | 25 |  |
| III | 18 | 40 |  | 24 | 34 |  | 41 | 17 |  |
| *Kruskal-Wallis tests, others chi-square tests |  |  |  |  |  |  |  |  |  |
