# Supplementary material for: Proposal of a grading system for squamous cell carcinoma of the lung — the prognostic importance of tumour budding, single cell invasion, and nuclear diameter
Source: Virchows Arch. 2023 Aug 9;483(3):393–404. doi: 10.1007/s00428-023-03612-8 (PMC10542270; doi:10.1007/s00428-023-03612-8)
Supplement: Supplementary file 4 — (DOCX 16 kb) [file 428_2023_3612_MOESM3_ESM.docx]

Article title: Proposal of a grading system for squamous cell carcinoma of the lung – the prognostic importance of tumour budding, single cell invasion, and nuclear diameter

Journal name: Virchows Archiv

Author names: Noémi Zombori-Tóth, Fanni Hegedűs, László Tiszlavicz, József Furák, Gábor Cserni, Tamás Zombori

Corresponding author: Tamás Zombori, MD, PhD; [zombori.tamas@med.u-szeged.hu](mailto:zombori.tamas@med.u-szeged.hu)

**Online Resource 3** Results of the univariate Cox proportional hazard model focusing on non-significant parameters in the multivariate model (HR: hazard ratio, 95%CI: 95% confidence interval).

|  | Overall survival | | | Recurrence-free survival | | |
| --- | --- | --- | --- | --- | --- | --- |
| Parameters | HR | 95%CI | *p* | HR | 95%CI | *p* |
| Age (years) |  |  |  |  |  |  |
| <64 | reference |  |  | reference |  |  |
| ≥64 | 1.22 | 0.59-2.50 | 0.581 | 0.93 | 0.54-1.59 | 0.798 |
| Gender |  |  |  |  |  |  |
| Female | reference |  |  | reference |  |  |
| Male | **2.84** | **1.02-8.14** | **0.049** | 1.72 | 0.88-3.34 | 0.107 |
| Smoking |  |  |  |  |  |  |
| Never | reference |  |  | reference |  |  |
| Ever | 1.11 | 0.44-2.45 | 0.452 | 1.09 | 0.51-1.89 | 0.71 |
| Surgery |  |  |  |  |  |  |
| Sublobar resection | reference |  |  | reference |  |  |
| Lobectomy | 0.56 | 0.25-1.25 | 0.167 | 1.15 | 0.54-2.44 | 0.713 |
| Adjuvant therapy |  |  |  |  |  |  |
| Absent | reference |  |  | reference |  |  |
| Present | 0.456 | 0.17-1.19 | 0.109 | 1.72 | 0.97-3.00 | 0.055 |
| Mitotic activity |  |  |  |  |  |  |
| Low | reference |  |  | reference |  |  |
| High | 0.88 | 0.39-1.98 | 0.761 | 1.19 | 0.63-2.20 | 0.606 |
| Pleural invasion |  |  |  |  |  |  |
| PL0 | reference |  |  | reference |  |  |
| PL1 | 1.12 | 0.34-3.61 | 0.845 | 2.51 | 0.75-8.33 | 0.132 |
| PL2 | 0.09 | 0.00-1.72 | 0.788 | 0.25 | 0.05-1.37 | 0.567 |
| PL3 | 0.49 | 0.12-2.05 | 0.336 | 1.15 | 0.27-4.89 | 0.842 |
| Vascular invasion |  |  |  |  |  |  |
| Absent | reference |  |  | reference |  |  |
| Present | 0.89 | 0.34-2.34 | 0.820 | 0.81 | 0.38-1.73 | 0.597 |
| Lymphovacular invasion |  |  |  |  |  |  |
| Absent | reference |  |  | reference |  |  |
| Present | 1.39 | 0.67-2.89 | 0.374 | 1.69 | 0.97-2.94 | 0.063 |
| Perineural invasion |  |  |  |  |  |  |
| Absent | reference |  |  | reference |  |  |
| Present | 0.57 | 0.17-1.90 | 0.367 | 0.81 | 0.38-1.72 | 0.584 |
