# Supplementary material for: Proposal of a grading system for squamous cell carcinoma of the lung — the prognostic importance of tumour budding, single cell invasion, and nuclear diameter
Source: Virchows Arch. 2023 Aug 9;483(3):393–404. doi: 10.1007/s00428-023-03612-8 (PMC10542270; doi:10.1007/s00428-023-03612-8)
Supplement: Supplementary file 7 — (DOCX 16 kb) [file 428_2023_3612_MOESM5_ESM.docx]

Article title: Proposal of a grading system for squamous cell carcinoma of the lung – the prognostic importance of tumour budding, single cell invasion, and nuclear diameter

Journal name: Virchows Archiv

Author names: Noémi Zombori-Tóth, Fanni Hegedűs, László Tiszlavicz, József Furák, Gábor Cserni, Tamás Zombori

Corresponding author: Tamás Zombori, MD, PhD; [zombori.tamas@med.u-szeged.hu](mailto:zombori.tamas@med.u-szeged.hu)

**Online Resource 5** The results of multivariate cox proportional hazards models (OS: overall survival, HR: hazard ratio, CI: confidence interval, STAS: spread through air spaces, RFS: recurrence-free survival)

| **OS - Regression I** | **HR** | **95%CI** | ***p*** |
| --- | --- | --- | --- |
| Histological subtype | 1.52 | 0.89-2.31 | 0.369 |
| Tumour budding | **4.61** | **1.38-15.32** | **0.013** |
| Single cell invasion | 1.87 | 0.91-3.23 | 0.149 |
| Nuclear diameter | **4.89** | **2.22-10.76** | **<0.001** |
| STAS | **2.34** | **1.12-4.86** | **0.023** |
| Stage | 0.97 | 0.78-1.78 | 0.132 |
| **OS - Regression II** | **HR** | **95%CI** | ***p*** |
| Histological subtype | 1.48 | 0.85-2.22 | 0.283 |
| Tumour budding extension (Weichert) | **2.52** | **1.45-4.38** | **<0.001** |
| Single cell invasion | 1.59 | 0.90-3.01 | 0.112 |
| Nuclear diameter | **5.30** | **2.41-11.65** | **<0.001** |
| STAS | **2.45** | **1.17-5.11** | **0.017** |
| Stage | 1.01 | 0.81-1.92 | 0.111 |
| **OS - Regression III** | **HR** | **95%CI** | ***p*** |
| Histological subtype | 1.60 | 0.93-2.35 | 0.148 |
| Single cell invasion | 1.59 | 0.90-3.01 | 0.112 |
| Kadota-grade |  |  |  |
| G1 | reference |  |  |
| G2 | 0.258 | 0.033-1.86 | 0.176 |
| G3 | **8.59** | **3.83-19.14** | **<0.001** |
| STAS | **3.37** | **1.59-7.14** | **<0.001** |
| Stage | 1.20 | 0.89-1.99 | 0.105 |
| **OS - Regression IV** | **HR** | **95%CI** | ***p*** |
| Histological subtype | 1.45 | 0.88-2.12 | 0.201 |
| Nuclear diameter | **5.19** | **2.36-11.4** | **<0.001** |
| Weichert-grade |  |  |  |
| G1 | reference |  |  |
| G2 | **4.94** | **1.14-21.33** | **0.032** |
| G3 | **10.92** | **2.33-41.24** | **<0.001** |
| STAS | **2.52** | **1.21-5.27** | **0.013** |
| Stage | 0.91 | 0.76-1.45 | 0.179 |
| **OS - Regression V** | **HR** | **95%CI** | ***p*** |
| Histological subtype | 1.23 | 0.81-2.34 | 0.356 |
| Grading proposal |  |  |  |
| G1 | reference |  |  |
| G2 | **6.34** | **1.21-16.42** | **0.03** |
| G3 | **15.89** | **2.66-38.73** | **<0.001** |
| STAS | **2.72** | **1.30-5.68** | **0.008** |
| Stage | 0.91 | 0.80-1.78 | 0.167 |
| **RFS - Regression I** | **HR** | **95%CI** | ***p*** |
| Single cell invasion | **2.48** | **1.34-4.62** | **<0.001** |
| Kadota-grade |  |  |  |
| G1 | reference |  |  |
| G2 | 0.39 | 0.133-1.14 | 0.085 |
| G3 | **2.44** | **1.13-5.26** | **0.023** |
| STAS | **3.28** | **1.89-5.67** | **<0.001** |
| Stage | 1.18 | 0.78-2.3 | 0.666 |
| **RFS - Regression II** | **HR** | **95%CI** | ***p*** |
| Nuclear diameter | **3.03** | **1.74-5.25** | **<0.001** |
| Weichert-grade |  |  |  |
| G1 | reference |  |  |
| G2 | 1.78 | 0.87-3.67 | 0.114 |
| G3 | **3.26** | **1.36-7.78** | **0.008** |
| STAS | **3.01** | **1.71-5.25** | **<0.001** |
| Stage | 0.98 | 0.67-1.56 | 0.649 |
| **RFS - Regression III** | **HR** | **95%CI** | ***p*** |
| Grading proposal |  |  |  |
| G1 | reference |  |  |
| G2 | **3.91** | **1.36-11.22** | **0.011** |
| G3 | **9.29** | **3.14-19.40** | **<0.001** |
| STAS | **3.51** | **1.91-5.72** | **<0.001** |
| Stage | 1.83 | 0.87-3.21 | 0.462 |
