# Supplementary material for: Proposal of a grading system for squamous cell carcinoma of the lung — the prognostic importance of tumour budding, single cell invasion, and nuclear diameter
Source: Virchows Arch. 2023 Aug 9;483(3):393–404. doi: 10.1007/s00428-023-03612-8 (PMC10542270; doi:10.1007/s00428-023-03612-8)
Supplement: Supplementary file 8 — (DOCX 13 kb) [file 428_2023_3612_MOESM6_ESM.docx]

Article title: Proposal of a grading system for squamous cell carcinoma of the lung – the prognostic importance of tumour budding, single cell invasion, and nuclear diameter

Journal name: Virchows Archiv

Author names: Noémi Zombori-Tóth, Fanni Hegedűs, László Tiszlavicz, József Furák, Gábor Cserni, Tamás Zombori

Corresponding author: Tamás Zombori, MD, PhD; [zombori.tamas@med.u-szeged.hu](mailto:zombori.tamas@med.u-szeged.hu)

**Online Resource 6** The results of interclass correlation (ICC: interclass correlation coefficient, CI: confidence interval)

|  | **ICC** | **95%CI** |
| --- | --- | --- |
| **Tumour budding score** | 0.80 | 0.75-0.84 |
| **Nuclear diameter score** | 0.79 | 0.73-0.84 |
| **Single cell invasion score** | 0.87 | 0.84-0.90 |
| **Grading proposal** | 0.88 | 0.84-0.91 |
